# Microleakage of Posterior Composite Restorations with Fiber Inserts Using two Adhesives after ging

**Published:** 2013-09

**Authors:** F Sharafeddin, H Yousefi, Sh Modiri, A Tondari, SR Safaee Jahromi

**Affiliations:** a Dept. of Operative Dentistry, Biomaterial Research Center, School of Dentistry, Shiraz University of Medical Science, Shiraz, Iran.; b Dentist; c Post Graduate Student in Operative Dentistry, School of Dentistry, Shiraz University of Medical Science, Shiraz, Iran.; d Post Graduate Student in Operative Dentistry, School of Dentistry, Shiraz University of Medical Science, Shiraz, Iran.; e Dept. of Operative Dentistry, School of Dentistry, Shiraz University of Medical Science, Shiraz, Iran.

**Keywords:** Microleakage, Polyethylene fiber, Composite restorations

## Abstract

**Statement of Problem:** Microleakage is one of the most frequent problems associated with resin composites, especially at the gingival margin of posterior restorations. Insertion of fibers in composite restorations can reduce the total amount of composite and help to decrease the shrinkage.

**Purpose:** The aim of this study was to evaluate the effect of polyethylene fiber inserts on gingival microleakage of class II composite restorations using two different adhesive systems.

**Materials and Method:** In this experimental study, class II cavities were prepared on 60 premolars. The gingival floor was located 1.0 mm below the CEJ. Dimension of each cavity were 3 mm buccolingually and 1.5 mm in axial depth. The specimens were divided into 4 groups according to the adhesive type and fiber insert (n=4). Single bond and Clearfill SE bond and Filtek p60 were used to restore the cavities. In groups without fiber inserts composite was adapted onto cavities using layering technique. For cavities with fiber inserts, 3 mm piece of fiber insert was placed onto the composite increment and cured. The specimens were stored in distilled water at 37^o^C for 6 months. All specimens were subjected to 3000 thermo-cycling. The tooth surfaces except for 1 mm around the restoration margins covered with two layers of nail varnish .The teeth were immersed in 2% Basic Fuchsin for 24 hours, then rinsed and sectioned mesiodistally. The microleakage was determined under a stereomicroscope (40X). Data were statistically analyzed by Kruskal-wallis and Mann-Whitney U tests (*p*< 0.05).

**Results:** The Kruskal-Wallis test revealed no significant differences in mean microleakage scores among all groups (*p*= 0.281).

**Conclusion:** Use of polyethylene fiber inserts and etch-and-rinse and self-etch adhesives had no effect on microleakage in class II resin composite restorations with gingival margins below the CEJ after 6- month water storage.

## Introduction

Glass and polyethylene fibers are used as reinforcing agent in composite restorations [1-2]. Currently, light cure resin composites are widely used to restore posterior teeth due to their esthetic properties and their ad-

hesion to tooth structures [3-4].

Microleakage is one of the most frequent encountered problems, especially at the gingival margin of class II restorations [5-6]. 

 Microleakage may lead to postoperative sensitivity, recurrent caries, marginal deterioration, pulp injury and enamel fracture [7].

Less polymerization shrinkage can be obtained if the total amount of composite material for restoration of a class II cavity is reduced [8]. Furthermore, different methods such as reducing the composite polymerization rate, using the incremental placement technique and reducing the C-factor have been suggested to decrease the microleakage of resin composite materials [5, 8]. 

Reinforcement of resin with fiber glass has improved mechanical and physical properties of resin composite materials [9].

To improve the mechanical properties of dental materials , new dental products such as glass , polyethylene ,quartz , carbon and other fibers have been made available recently [8].

Fiber-reinforced composites have a wide range of application such as periodontal splints and fixed partial dentures in dentistry [10].

High modulus of elasticity and low flexural modulus of polyethylene fibers modify the interfacial stresses developed along the etched enamel- resin boundary [11].

Inserting of polyethylene fibers in composite restoration can reduce the total amount of resin matrix required for restoration and decrease the shrinkage and microleakage [5, 8].

The aim of this study was to evaluate the effect of polyethylene fiber inserts on gingival microleakage of class II resin composite restorations by using two different bonding systems.

## Materials and Method

The manufacturers and the composition of the material used in current study are shown in table 1. 

In this experimental study, 60 intact human premolars were cleaned with periodontal scalers and rotary brushes. The teeth were then mounted in acrylic bases, up to 2mm apical to the CEJ.

**Figure 1:**
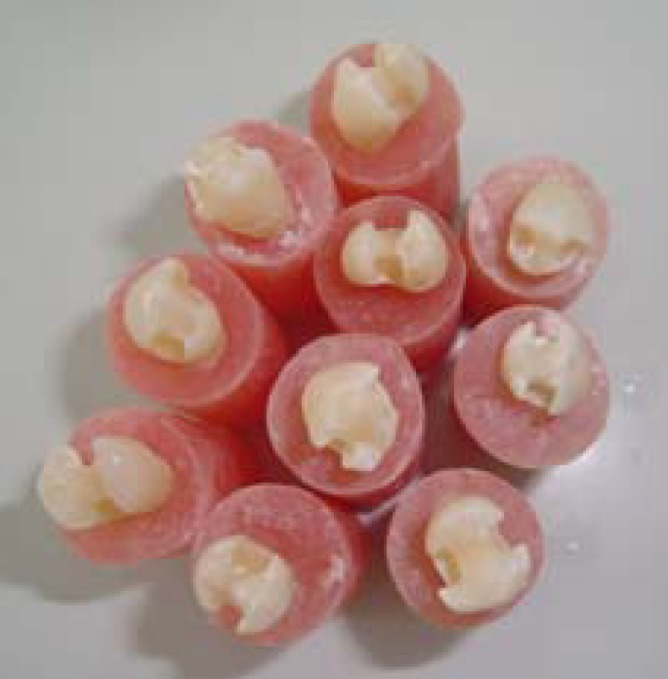
Premolars with completed slot preparation

Then class II slot cavities were prepared on both proximal sides of each premolars using a 245 tungsten carbide bur (SS White; Great White Series, LAKEOOD, NJ, USA) in a water-cooled high-speed air turbine handpiece .All line angles were rounded. The gingival floor of the slot cavities was located at least 1.0 mm below the CEJ on the root surface. Each slot was 3 mm buccolingually wide and 1.5 mm in axial depth (Figure 1). The dimension of the cavities were verified with a periodontal probe. The teeth were randomly divided into 4 groups (n=4).

A universal metal matrix band/retainer (Tofflemire) was placed around each prepared tooth. Each cavity was cleaned with water spay and air-dried. The bonding agent assigned to each group were applied according to manufacturer’s instructions (Table 1).

A posterior resin composite (Filtek p60; shade A2, 3M ESPE) was used to restore all cavities. The specimens were divided into 4 groups according to the adhesive type and fiber insert (Table 2). The cavities without fiber inserts were used as controls .An approximate 2 mm- layer of p60 was adapted onto gingival floor and light activated for 40 seconds, using a halogen curing unit (400 mW/cm2; Unicorn Med., Korea). A second layer was added diagonally on one side and light polymerized for 40 seconds. Two other increments, filling the remainder of the box, were placed and similarly light polymerized.

**Table 1:** Products used in this study

**Products**	**Compositions**	**Manufacturer**
Filtek p60	Triethylenglycol dimethacrylate, urethane dimethacrylate, silica, zirconium bisphenylethylenemethacrylate 84.5%, 0.6 mm	3M ESPE Dental product
Single bond	Etching gel: phosphoric acid (35%), colloidal silica thickener, color, water	3M ESPE Dental product
	Adhesive: polyalkenoic acid, hydroxyrthylmethacrylate, bisphenol A diglycidyl ether dimethacrylate, dimethacrylate copolymer, ethanol, water	
Clearfil SE bond	Primer: hydroxyethylmethacrylate, methacryloloyloxydecyl dihydrogen phosohate, hydrophilic dimethacrylate, DL-camphorquinone, N-diethanol-p-toluidine, water	3M ESPE Dental product
	Adhesive: hydroxyethylmethacrylate,bisphenyl glycidylmethacrylate, methacryloloyoxydeyl dihydrogen phosphate, hydrophilic dimethacrylate, DL-camphorquinone, N-diethanol-p-toluidine, silinated colloidal silica (10 % ,microthin)	
Ribbond- THM	Polyethylene fiber	Ribbond-THM
Resist	Bis-GMA,alkali-soluble polymers,fluorinated ester-containing units	bTd

**Table 2:** Distribution of the experimental groups among the two bonding agent and fiber insertion

**Groups**	**Bonding agent**	**Fiber insert**
G1	Single bond	--------
G2	Single bond	Polyethylene fiber
G3	Clearfil SE bond	--------
G4	Clearfil SE bond	Polyethylene fiber

For cavities with fiber inserts, a less than 1- mm- thick resin composite was first placed on the gingival floor. Then, a 3- mm piece of fiber insert was preimpre-gnated in Resist (NSI Dental Pty Ltd; Australia) for 5 minutes and then placed onto the composite increment and condensed through it to be adapted against the gingival floor and light-polymerized for 40 seconds from the occlusal aspect. Three other diagonal layers of resin composite were placed and polymerized as stated previously.

Only the occlusal surfaces were then finished with 30-bladed tungsten carbide bur (H 135 UF, H 379 UF. H 246 LUF; Brasseler, USA) in a high-speed handpiece with water cooling. Polishing was performed afterwards by an aluminum oxide point (Jiffy points; Ultradent).the specimens were then stored in distilled water at 37 ^0^C for 6 months in an incubator (Behdad; Iran). All specimens were then subjected to 3000 thermo-cycling between 5 ^0^C and 55 ^0^C in water bath (SANAF; Iran) with dwell time of 30 seconds. Tooth surfaces except for 1mm around the restoration margins were covered with two layers of nail varnish. The teeth were then immersed in 2% Basic Fuchsin for 24 hours, then rinsed in tap water for 5 minutes. Then each tooth was sectioned mesiodistally with a high-speed diamond saw (Isomet; buchler, USA). The section with deepest penetration was selected to be reported in our study. 

Microleakage was determined blindly by three observers under a stereomicroscope with 40X magnification according to a six-point scale:

0= no leakage

1= leakage extending to the outer half of the gingival floor

2= leakage extending to the inner half of the gingival floor

3= leakage extending through gingival floor up to 1/3 of the axial wall

4=leakage extending through gingival floor up to 2/3 of the axial wall

5=leakage extending through the gingival wall up to the DEJ level

Data were statistically analyzed by non-parametric Kruskal-Wallis test and Mann-Whitney U test (*p*< 0.05).

## Results

**Figure 2:**
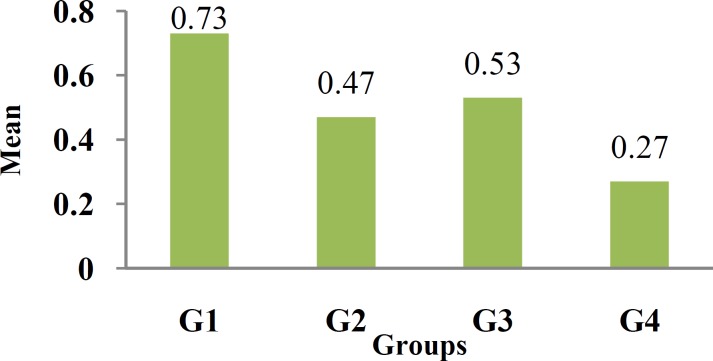
Mean of microleakage

The mean and standard deviation of microleakage scor-es for all groups are presented in table 3 and figure 2. 

G1: Single bond G2: Single bond + polyethylene fiber

G3: Clearfil SE bond G4: Clearfil SE bond + polyethylene fiber

**Table 3:** Microleakage scores distribution among the test groups with means and standard deviations

**Groups**		**Microleakage scale**						**Mean**	**SD**
		**Percent**							
		**0**	**1**	**2**	**3**	**4**	**5**		
Adper single bond	G1	7	8	0	0	0	0	0.73	0.799
		46.7%	53.5%	0.0%	0.0%	0.0%	0.0%		
	G2	9	6	0	0	0	0	0.47	0.640
		60.0%	40.0%	0.0%	0.0%	0.0%	0.0%		
Clearfil SE bond	G3	8	7	0	0	0	0	0.53	0.640
		53.3%	46.7&	0.0%	0.0%	0.0%	0.0%		
	G4	12	3	0	0	0	0	0.27	0.594
		80.0%	20.0%	0.0%	0.0%	0.0%	0.0%		

G1: Single bond           G2: Single bond + polyethylene fiber           G3: Clearfil SE bond          G4: Clearfil SE bond + polyethylene fiber

The Kruskal-Wallis test revealed no significant differences in mean microleakage scores among the groups (*p*= 0.281). Mann-Whitney test showed that Group 1 had higher microleakage scores than the Group 2, but this different was not significant (*p*= 0.412). Group 4 had lower scores than group 2 but this difference was not statistically significant (*p*= 0.278). Group 3 had higher scores in terms of microleakage but it was not statistically significant (*p*= 0.161). Group 1 had higher microleakage than group 3, but again this difference was not statistically significant (*p*= 0.102).

Generally, specimens from the groups with polyethylene fiber inserts had lower scores than the groups without inserts, but this difference was not statistically significant.

Clearfil SE bond produced the lower degree of microleakage than those with Adper single bond application, but these differences were not statistically significant.

The lowest microleakage scores belonged to group 4 (Clearfil SE bond) and the highest scores were seen in group 1 (Adper single bond) but it was not statistically significant.

## Discussion

One of the most important clinical drawbacks of the resin composite restorative materials is their marginal microleakage [12]; which occurs as a result of polymerization shrinkage, fatigue-cycling, thermal changes in oral environment [13].

Fibers have been added to resin composites to solve this problem by reducing the total amount of composite and increasing the resistance of initial composite increment against pull- away from the gingival margin toward the light curing unit [4, 8, 14].

In the current study groups, polyethylene fiber inserts did not show significant reduction in microleakage. This finding is in contrast with some previous studies, suggesting that fiber insertion could reduce marginal microleakage [4-5, 8]. This finding can be related to the layering technique that was used in the current study.

It has been reported that glass fibers were more effective than polyethylene fibers for reinforcement due to their good adhesion to the resin matrix [14].However, in our study, we used only polyethylene fiber that was as effective as composite restorations without fiber inserts.

Another approach to improve marginal integrity and consequent clinical problems is the use of lining materials with low viscosity such as glass ionomers or some types of bonding agents [15-16]. We used one layer of composite at the base of restorations in gingival floor which could reduce the polymerization shrinkage, occurring in bulk technique restorations at the base of box on gingival floor [17]. 

In the current study; two adhesive systems (two-step etch-and-rinse and one mild self-etching primer) were used to evaluate the effect of type of bonding agent on marginal microleakage.

The bonding mechanism of these two systems is quite different [18]. The bonding mechanism of etch-and-rinse system is diffusion-based, the way of resin infiltrates into collagen fibrils and forms hybrid layer via micromechanical bonding [19]. In self-etching system, the bonding mechanism is based on the dissolution of the smear layer and penetration of acidic monomers in underlying dentin which leads to the hybrid layer formation [20]. In mild self-etching adhesive systems, some hydroxyapatite remain around the collagen fibrils caused by low acidity of monomers and may have chemical reaction with functional monomer in addition to micromechanical retention which can reduce marginal microleakage [18]. The results of this study showed that these two bonding systems have similar sealing ability in the margins of restorations, which is consistent with some previous researches [20-22]. 

There are different methods to evaluate microleakage, such as scanning electron microscopy, electro-chemical studies and dye penetration [23-24]. Dye penetration, a semi-quantitative method, was used in current study. Some studies have reported that there was no difference between these methods regarding the evaluation of microleakage [18, 20]. 

In the current study, all specimens were stored in water for 6 months which was different from previous studies that evaluated microleakage of class II composite restoration with fiber insert.

One study showed that 3- month- storage had no effect on microleakage [25] but some other studies concluded that storage time increases the microleakage in some bonding systems [26-28]. It seems that fiber insertion or application of different bonding systems have no effect on the gingival microleakage of class II resin composite restorations after 6 months of storage .It may be due to the equal hybridization of self- etch and total- etch adhesives after 6 months. Therefore,the microleakage of restorations with or without fiber was equal.

In this study, we evaluated microleakage of gingival margins because most previous studies reported that the microleakage of gingival margins in composite restorations was more than the microleakage of occlusal margins [3-4]. Bleaching agent may affect the physical and mechanical properties of composite resins [29]. 

Glass fibers and low- shrinkage composites, like silorane-based composite, can also be evaluated, as a suggestion for future studies, to reduce the marginal microleakage of fiber reinforced composite restorations, by employing different techniques and evaluation in different media.

## Conclusion

Within the limitation of the current in vitro study it can be stated that:

1. The use of polyethylene fiber inserts have no effect on microleakage in class II resin composite restorations with gingival margins below the cemento-enamel junction after 6- month-storage in water
2. There is no difference between two bonding systems (two-step etch-and-rinse and self-etching primer) in marginal microleakage in class II composite restorations after 6-month-storage in water.
